# Hybridisation as a Potential Extinction Threat to an Endangered Australian Frog

**DOI:** 10.1002/ece3.71494

**Published:** 2025-05-29

**Authors:** Gracie Liu, Jodi J. L. Rowley

**Affiliations:** ^1^ Australian Museum Research Institute, Australian Museum Sydney New South Wales Australia; ^2^ Centre for Ecosystem Science, School of Biological, Earth and Environmental Sciences UNSW Sydney Sydney New South Wales Australia

**Keywords:** amphibian, Australia, hybrid zone, interspecific hybridisation, introgression, sympatric

## Abstract

Of the many threats to species' survival, genetic threats such as hybridisation and introgression are often overlooked. Threatened or range‐restricted species that hybridise with more abundant and widespread relatives can be particularly vulnerable to declines or extinction via demographic or genetic swamping. Conservation of these species requires detection of hybridisation, but this can be difficult when hybrids are morphologically indistinguishable from parental species (i.e., cryptic). We used single nucleotide polymorphism data to examine hybridisation and introgression between the endangered Booroolong frog ( *Litoria booroolongensis* ) and the more abundant eastern stony creek frog ( *Litoria wilcoxii* ), two Australian stream frog species not previously reported to hybridise. To assess whether hybrids and parental individuals could be identified by phenotype, we compared morphological and genotypic classifications of individuals. Genotyping revealed relatively high rates of hybridisation and introgression (19% (24/126) were F1 or F2 hybrids, or backcrosses) in the New South Wales Central Tablelands. Hybrids were present in all sites where the species were syntopic (five of seven sites), suggesting that hybridisation is constrained primarily by a lack of opportunity. Within these sites, the median rate of hybridisation was 31.8% (range: 5.3–100%). Based on the likely extent of syntopy, hybridisation is plausible across more than 70% of the geographic range of *L. booroolongensis* , with potentially negative consequences for the species' persistence. Concerningly, only 42% of hybrids were correctly identified by morphology. Our results emphasise the need for genetic data to accurately distinguish hybrids and suggest that hybridisation could be occurring undetected between many related species, representing a potentially insidious threat to threatened and range‐restricted species. Conservation policies will need to consider the possibility of hybridisation and evaluate its consequences to appropriately manage and prevent further declines of threatened species.

## Graphical abstract

We used single nucleotide polymorphism data and morphological assessment to investigate hybridisation and introgression between two Australian frog species not previously reported to hybridise, the endangered Booroolong frog ( *Litoria booroolongensis* ) and its abundant congener, the eastern stony creek frog ( *Litoria wilcoxii* ). Hybrids could not be reliably identified by phenotype, but genotyping revealed relatively high rates of hybridisation and introgression. Our results emphasise the need for genetic tools to detect cryptic hybrids and suggest that hybridisation could be occurring undetected between many related species, representing a potentially insidious threat to threatened and range‐restricted species.

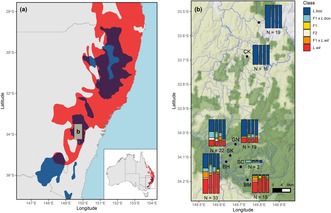

## 1 Introduction

Managing biodiversity often requires mitigating the effects of multiple threatening processes, including habitat loss and modification, introduced species, disease, climate change, pollution and overexploitation (Kingsford et al. 2009). Genetic threats such as hybridisation and introgression are frequently overlooked but can potentially cause species declines or extinctions (Draper et al. 2021). This can occur via genetic swamping, where hybrids partially or, in extreme cases, completely replace pure parental genotypes, or via demographic swamping, where the parental species waste reproductive efforts producing unfit hybrids (Kleindorfer et al. 2014; Ottenburghs 2021; Todesco et al. 2016). However, hybridisation may also be a useful conservation management tool for at‐risk species, as it can increase genetic diversity, the transfer of adaptations and, in some circumstances, rescue inbred populations (Chan et al. 2019; Draper et al. 2021; Frankham 2015; Vedder et al. 2022). Because the outcomes of hybridisation vary widely and are highly context‐dependent, it is important to identify affected taxa and assess the impacts on species persistence.

Hybridisation and introgression are relatively common across taxa, including plants (Fogelqvist et al. 2015; Hoban et al. 2012), invertebrates (Dlouhá et al. 2010), fish (Couch et al. 2016; Coulter et al. 2020; Lewis et al. 2021), reptiles (Haines et al. 2016), birds (Lontkowski and Maciorowski 2010), mammals (Mallet 2005) and amphibians (Beebee 2005; Ottenburghs 2021). An estimated 25% of the plant species and 10% of the animal species hybridise in the wild (excluding hybridisation with non‐native species), but hybridisation rates can be much higher within certain taxa (e.g., 76% of the British duck species; Mallet 2005). Though amphibians were not evaluated in these estimates, many amphibian species are known to hybridise naturally (e.g., Borzée et al. 2020; Donnellan et al. 2023; Ficetola et al. 2019; Hoskin 2007; Mahony et al. 2021; O'Brien et al. 2018; Parkin et al. 2023; Payne 2014; Smith et al. 2013).

Hybridisation is typically not a conservation concern when it occurs infrequently and is restricted to narrow contact zones (Borzée et al. 2020; Hoskin 2007). However, genetic and demographic swamping can be a risk when threatened or range‐restricted species hybridise with more abundant species (Costa et al. 2017; Georges et al. 2018; Lewis et al. 2021) and when hybridisation is frequent and spatially extensive relative to the hybridising species' range (Borzée et al. 2020). Human activities can promote these conditions through, for example, habitat modification (Grabenstein and Taylor 2018; Ottenburghs 2021) or translocations of non‐native species (unintentionally or deliberately, for example, for genetic rescue; Frankham 2015; or for fishing and hunting; Johnson et al. 2010, Barilani et al. 2007). These activities can break down geographic, physical and temporal reproductive barriers, bringing previously isolated species or populations into contact, enabling hybridisation (Grabenstein and Taylor 2018, Ottenburghs 2021), even between evolutionary divergent taxa (Aplin et al. 2024; Georges et al. 2018).

Landscape changes can increase suitable habitat for certain species while decreasing the habitat of others and may cause species to expand their range into the range of others (Borzée et al. 2020; Okes et al. 2008). This can provide opportunities for hybridisation across a large proportion, or even the entirety, of a species' range and may be coupled with shifts in species abundance, with some species declining and some increasing or persisting at high abundance (Borzée et al. 2020; Didham et al. 2007). When sympatric species that are capable of hybridisation have unbalanced population sizes (i.e., one is more abundant than the other) and reproductive isolation is partly density dependent, demographic and genetic swamping may threaten the rarer species (Field et al. 2008; Hoskin 2007). In addition, disturbance or homogenisation of habitat structure can erode prezygotic barriers formed by species' different microhabitat preferences (e.g., separate calling locations; Bell and Irian 2019) or breeding phenology (Lamont et al. 2003). Habitat modifications can also disrupt visual, chemical or auditory signals that normally promote species recognition and prevent hybridisation (Ottenburghs 2021). Together, these factors can increase hybridisation events, potentially to the detriment of one or both species.

For effective conservation of threatened species, it is important to identify hybrids and patterns of introgression and to consider their impacts alongside more widely recognised threatening processes. Frequent undetected hybridisation and introgression could undermine the accuracy of monitoring efforts (e.g., population estimates, distribution mapping; Dufresnes et al. 2017, Georges et al. 2018), hindering the development of appropriate conservation policies. Recent genetic tools have enabled accurate detection of hybrids and introgressed individuals (McFarlane and Pemberton 2019; Melville et al. 2017; Twyford and Ennos 2012) but hybrids are still regularly inferred from morphology (Lontkowski and Maciorowski 2010; Payne 2014). The latter can be a quick, inexpensive and non‐invasive means of detecting hybridisation in the field and is useful when hybrid traits are intermediate (Buck et al. 2020; Dlouhá et al. 2010) or unusual compared to the parental species (Neri et al. 2017), but hybrids are often cryptic, particularly in systems with extensive backcrossing. Hybrids may be morphologically similar or even indistinguishable from one of the parental species (Costa et al. 2017; Jasińska et al. 2010; Majtyka et al. 2022; Neri et al. 2017), rendering their morphological identification inaccurate and unreliable, especially when the parental species' traits are highly variable and overlap (Buck et al. 2020; Fogelqvist et al. 2015). Improving the detection of hybridisation requires evaluation of the efficacy of morphology‐based identifications for specific parental species and their hybrids.

**FIGURE 1:**
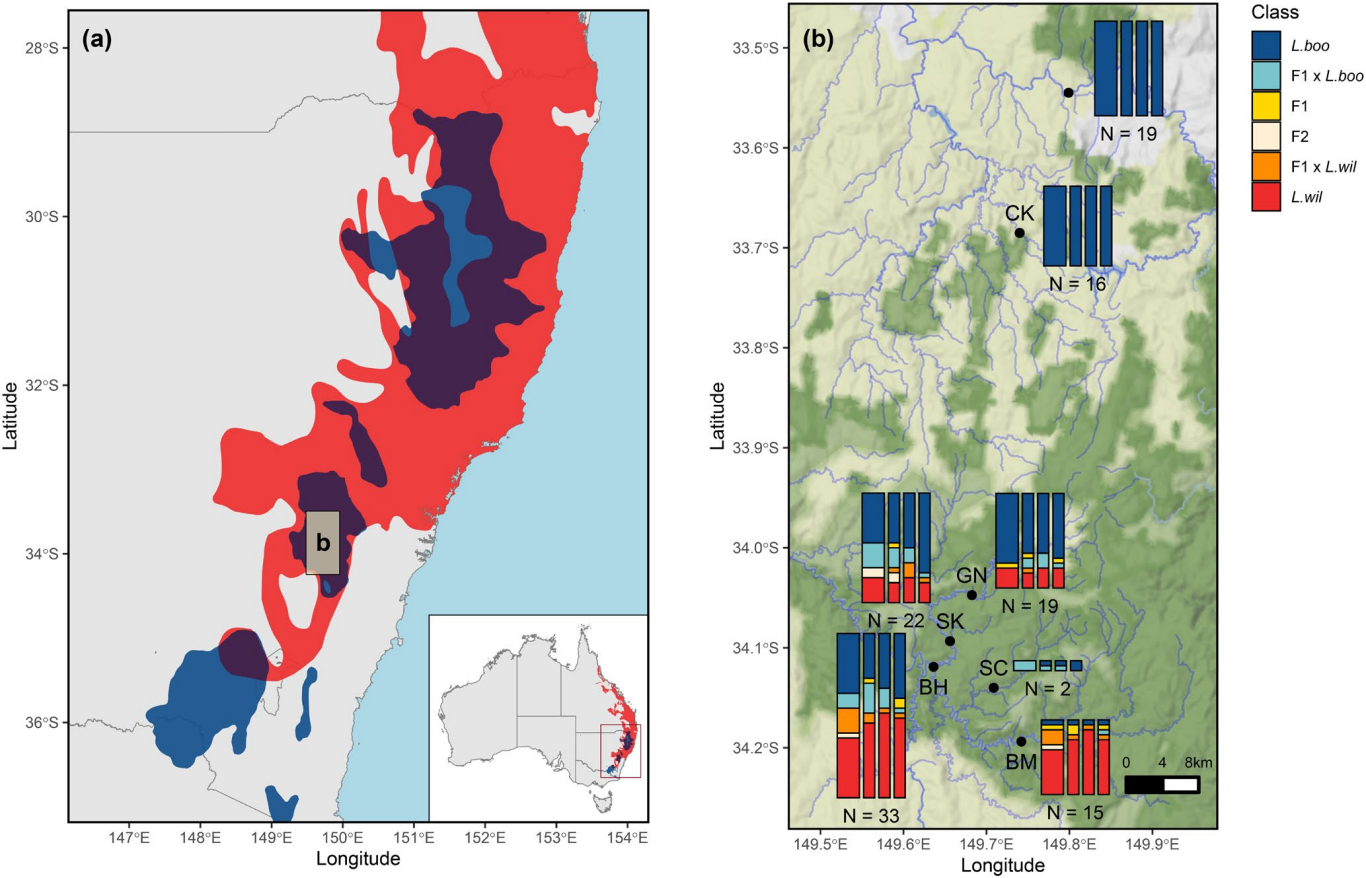
(a) Geographic distribution of *Litoria booroolongensis* (dark blue), *Litoria wilcoxii* (red) and overlap zones (dark purple) in New South Wales, Australia (Cutajar et al. 2022a), with inset showing the location in Australia; rectangle labelled ‘b’ indicates the location of the study sites, shown in detail in panel b. (b) Zoomed map of study sites (black points) and sample sizes (*N*), with column heights proportional to the number of frogs assigned to each class (parental *L. booroolongensis* (*L. boo*); parental *L. wilcoxii* (*L. wil*); F1 hybrids (F1); F2 hybrids (F2); and backcrosses of F1 hybrids to one of the parentals (F1 × *L. boo* or F1 × *L. wil*)) at each site based on genetic data (left column; NewHybrids classification) and morphology (narrower columns to the right; each column represents one validator).

The Booroolong frog ( *Litoria booroolongensis* ) and the eastern stony creek frog ( *Litoria wilcoxii* ) are closely related, but morphologically and genetically distinct, sympatric stream‐breeding frog species (fixed differences at ≥ 11% of their loci; Donnellan and Mahony 2004). Although a more comprehensive examination of their phylogenetic relationship is required, *Litoria booroolongensis* is believed to be paraphyletic with the *Litoria lesueuri* species group, which includes *Litoria wilcoxii* (Donnellan and Mahony 2004; Hutchinson and Maxson 1986). Hybridisation has not been documented between the species, but *L. booroolongensis* is often found syntopically with *L. wilcoxii* and drastic changes to the species' population size and relative abundances in recent decades raise the possibility of widespread interspecific interactions and undetected hybridisation and introgression. *Litoria wilcoxii* is often locally abundant across its broad eastern Australian distribution (Portway et al. 2020) and is listed as Least Concern under the International Union for Conservation of Nature (IUCN) Red List (Stuart 2006). In contrast, *L. booroolongensis* (Endangered under the Environment Protection and Biodiversity Conservation Act 1999; Critically Endangered under the IUCN Red List; Hero et al. 2004) has a smaller disjunct distribution within New South Wales (NSW) and Victoria (Figure 1a). The species has declined rapidly since the mid‐1980s (Rowley and Cutajar 2018) and has been extirpated from more than half of its historic range, likely due to the combined impacts of habitat loss and modification (Gillespie and Hines 1999; Hunter and Smith 2013), disease (Hunter et al. 2018; Portway et al. 2020), drought (McFadden et al. 2010) and predation by invasive fish (Hunter et al. 2011).

In its northern range, *L. booroolongensis* was once one of the most abundant frog species, outnumbering *L. wilcoxii* (Heatwole et al. 1995), but is now uncommon and persists in few sites (Portway et al. 2020; Rowley and Cutajar 2018). Meanwhile, *L. wilcoxii* has likely increased and now outnumbers *L. booroolongensis* in many sites, a change that may be reflected across other parts of its range (Portway et al. 2020). Yet, interspecific hybridisation and introgression and potential associated threats to the species' persistence remain unexplored.

We examined potential hybridisation between *L. booroolongensis* and *L. wilcoxii* . Specifically, we aimed to determine: (1) whether hybridisation and introgression occur between *L. booroolongensis* and *L. wilcoxii* ; and if so, (2) the frequency of hybridisation and introgression; (3) regions of syntopy where hybridisation may occur; and (4) whether the two species and their hybrids can be accurately distinguished by morphology by comparing morphological and genetic classification of individuals.

## 2 Materials and Methods

### 2.1 Study Sites and Sampling

During surveys in the Central Tablelands of New South Wales, Australia (Figure 1b), between March 2020 and March 2021, we detected both *L. booroolongensis* and *L. wilcoxii* , as well as several putative hybrids, which exhibited intermediate morphology or a mixture of the two species' traits. We sampled 126 of these individuals from across seven sites: five in Abercrombie River National Park and State Conservation Area (three along Retreat River: ‘The Glen’: GN [34.0° S, 149.7° E], ‘The Sink’: SK [34.1° S, 149.7° E] and ‘The Beach’: BH [34.1° S, 149.6° E]; one along Silent Creek: SC [34.2° S, 149.7° E]; and one along Abercrombie River: ‘Bummaroo Ford’: BM [34.2° S, 149.7° E]); one site in Essington State Forest (Captain Kings Creek: CK [33.7° S, 149.7° E]); and one site on crown and private land (Fish River: FH [33.6° S, 149.8° E]). Given that lack of prior documentation of hybridisation between the species, we focused sampling within the Central Tablelands because both species were expected to occur in the region at reasonable numbers to enable adequate sampling. Thus, if interspecific hybridisation and introgression was occurring, it would likely be detected in this region, with the results informing whether further examination of hybridisation across the remainder of the species' ranges (Figure 1a) is warranted.

We sampled individuals as they were encountered, without specifically targeting hybrids or individuals of one species or the other, and thus the relative numbers of *L. booroolongensis, L. wilcoxii*, and their hybrids likely reflect the population genetic structure at the sites. We measured mass and snout‐vent length (SVL) of each frog, collected a toe‐tip for genotyping and photographed the dorsal, lateral (left and right) and ventral surfaces for morphological identification, gently extending the legs to expose the side of the face and the inner and back of the thighs. Toe‐tips were placed in 100% ethanol and transferred to −20°C storage at the conclusion of each survey.

### 2.2 Molecular Analyses

Toe‐tips were sent to Diversity Arrays Technology (DArT Pty Ltd., Canberra, ACT, Australia; Kilian et al. 2012) for DNA extraction and sequencing. Sequencing for single nucleotide polymorphism (SNP) genotyping was achieved using DArTseq 1.0, which uses a combination of complexity reduction methods, fragment size selection and next‐generation sequencing (NGS) platforms (Kilian et al. 2012). For a detailed description of the PCR amplification, DNA sequencing, sequence processing and data generation methods, see Kilian et al. (2012) and Mahony et al. (2021).

Briefly, genome reduction is performed by a combination of restriction enzymes that target low‐copy DNA areas and is optimised for each organism, enabling detection of many informative SNPs across the genome (Melville et al. 2017). Here, DNA samples were processed in restriction enzyme digestion/ligation reactions using a combination of the PstI/SphI restriction enzymes. Polymorphic SNP markers within the samples were identified from sequences processed using proprietary DArT analytical pipelines (Kilian et al. 2012). The average read depth across loci was 9.2 reads per individual per locus for reference alleles and 7.3 for SNP alleles. DNA sequences and statistics (read depth, polymorphism information content, call rate, heterozygosity and reproducibility) are accessible from DArT Pty Ltd., Canberra, Australia (Report DLit22‐6707).

The SNP data received from DArT Pty Ltd. were read into a genlight object to facilitate processing and filtering with the ‘dartR’ package (Gruber et al. 2018) in R version 4.1.2 (R Core Team 2019). Only loci with 100% repeatability (reproducibility) were kept for subsequent analysis (Georges et al. 2018; Mahony et al. 2021; Parkin et al. 2023). We further filtered out loci with a call rate of < 98%, individuals with a call rate of < 70% and all monomorphic loci (Georges et al. 2018). Where there was more than one SNP per sequence tag, all but one SNP was removed at random (Georges et al. 2018; Mahony et al. 2021; Parkin et al. 2023).

To identify pure and hybrid individuals, we first visualised the genetic similarity among individuals using principal component analysis (PCA) ordination, implemented via the ‘gl.pcoa’ function in dartR. PCA ordination reduces complex high‐dimensional data (e.g., SNP datasets) into a few key dimensions that capture the most variation in the data, enabling visual assessment of potential hybrids. We examined the scree plot to determine the most appropriate number of principal components based on the percentage of variance explained. For a quantitative assessment, we analysed the data with NewHybrids software (Anderson and Thompson 2002), implemented in the ‘dartR’ package, to identify pure individuals, F1 and F2 hybrids and backcrosses of F1 hybrids to one of the parental species. NewHybrids, a Bayesian model‐based clustering method, uses Markov chain Monte Carlo (MCMC) simulations to estimate the posterior probability of an individual belonging to each of the defined genotypic classes (parental species A, parental species B, F1 hybrid, F2 hybrid and F1 backcrosses; Anderson and Thompson 2002). As the number of loci in our filtered dataset exceeded the maximum of loci (i.e., 200) analysable in NewHybrids, we selected 200 of the most informative loci for assessing hybridisation using the ‘AvgPIC’ method in the ‘gl.nhybrids’ function of dartR (Georges et al. 2018; Mahony et al. 2021; Parkin et al. 2023).

### 2.3 Potential Hybridisation Zones

To identify potential regions of hybridisation between *L. booroolongensis* and *L. wilcoxii* , we identified potential regions of syntopy from the most up‐to‐date and accurate distribution maps of the two species (Cutajar et al. 2022a), representing their known and presumed distributions (including regions where the species has been extirpated but which remain suitable habitat; Cutajar et al. 2022b) and calculated the area of overlap using the ‘sf’ package (Pebesma 2018) in R (version 4.1.2; R Core Team 2019). The overlapping regions therefore reflected habitat suitable for both species and represented regions where hybridisation may be occurring presently or could occur in the future (e.g., if reproductive barriers are weak and the species were to come into contact due to one or both species recolonising previously occupied areas).

### 2.4 Morphological Classification

To determine whether hybrids could be accurately identified by morphological assessment, three validators with experience in identifying *L. booroolongensis* and *L. wilcoxii* (i.e., species experts and field biologists) viewed photographs of each frog (dorsal, left and right lateral, and ventral surfaces, e.g., Figure 4) and independently classified them into one of six classes: pure *L. booroolongensis* ; pure *L. wilcoxii* ; F1 hybrid; F2 hybrid; backcross of F1 to *L. booroolongensis* ; or backcross of F1 to *L. wilcoxii* . Classification into these six classes enabled direct comparison with the results of the NewHybrids analyses. Recognising that it was probably unlikely that validators would be able to reliably distinguish between the various hybrid classes due to having no a priori knowledge of their morphology, we also combined all individuals that were thought to belong to each hybrid class (i.e., putative F1, F2 and backcrossed individuals) into one general hybrid category. This allowed us to determine whether validators could accurately distinguish between hybrids and non‐hybrids and whether further classification into hybrid categories might also be possible via morphological examination.

To assist with classification, validators were provided with reference dorsal, lateral and ventral photos (including side of face, inner thighs and back of thighs) of *L. booroolongensis* and *L. wilcoxii* and a table describing morphological characteristics of each species (including dorsum, groin, back of thighs, iris, head shape, tympanum, ventral surface and toe webbing; see Zenodo repository). Validators were free to consult other references (e.g., field guides), if desired. To minimise potential validator biases and ensure that validators could not use information about the collection sites, sample name or sampling order to inform their decisions, we assigned a random number to each frog that was independent from this information.

### 2.5 Data Analyses

We compared the morphological and genetic classification results in three ways. First, to determine whether the population composition (i.e., the proportion of pure and hybrid individuals of each class) could be accurately determined by morphology, we calculated the number and proportion of frogs that were classified into each parental and hybrid class based on morphology (averaged across validators) and based on genotype (NewHybrids results). To determine whether these significantly differed, we performed a chi‐squared test with the morphological classifications as the observed values and the genetic classifications as the expected values. Second, for all sampled *L. booroolongensis* , *L. wilcoxii* and their hybrids, we determined how many individuals were correctly identified by validators. We calculated the number and percentage of correct morphological identifications for each parental and hybrid class, assuming the NewHybrids classifications reflected the identity of the individuals. Third, we determined the accuracy of each validator's morphological classifications. For all frogs that were determined by morphology to be *L. booroolongensis* , *L. wilcoxii* or hybrids, we calculated the number and percentage that were correct, again assuming the NewHybrids classifications reflected the identity of the individual. In this instance, accuracy was based on each validator's morphological classifications so if a validator did not classify any frogs into a certain class, there was no measure of accuracy associated with that class. All analyses were performed in R version 4.1.2 (R Core Team 2019), relying on the tidyverse workflow (Wickham et al. 2019). The following R packages were also used for data visualisation: ‘ggmap’ (Kahle and Wickham 2013), ‘ggsn’ (Baquero 2019), ‘gridExtra’ (Auguie 2017) and ‘cowplot’ (Wilke 2020).

## 3 Results

### 3.1 Potential Hybridisation Zones

The geographic distributions of *L. booroolongensis* and *L. wilcoxii* overlapped across 58,202 km^2^, representing 70% and 17% of the known and predicted range of *L. booroolongensis* (82,562 km^2^) and *L. wilcoxii* (349,005 km^2^), respectively. Overlapping regions occurred across most of the Central and Northern Tablelands and the northern part of the Southern Tablelands (Figure 1a).

### 3.2 Molecular Analyses

**FIGURE 2:**
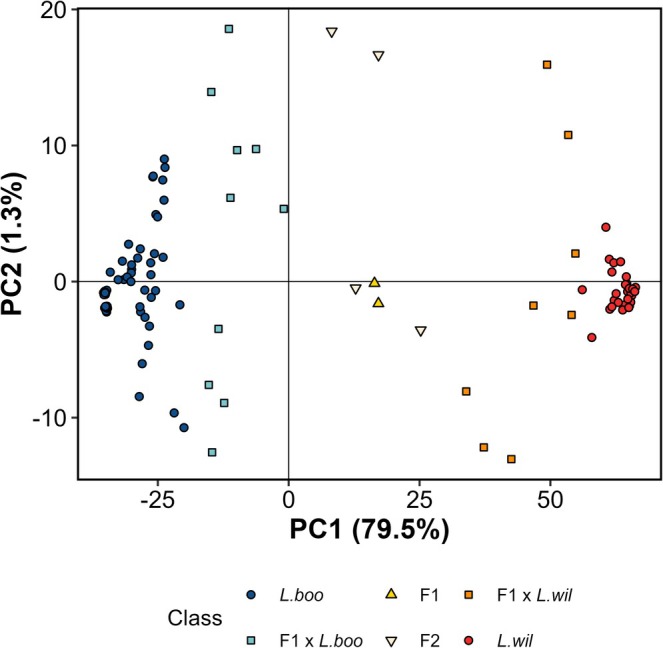
Plot of genetic similarities among individuals, showing first two dimensions (PC1 and PC2). Individuals were assigned to classes (parental *Litoria booroolongensis* (*L. boo*); parental *Litoria wilcoxii* (*L. wil*); F1 hybrids (F1); F2 hybrids (F2); and backcrosses of F1 hybrids to one of the parentals (F1 × *L. boo* or F1 × *L. wil*)) using NewHybrids software (Anderson and Thompson 2002).

A total of 131,064 polymorphic SNP loci were scored for 126 individuals of *L. booroolongensis* and *L. wilcoxii* . After filtering, 13,226 SNP loci remained. The PCA revealed two main groups corresponding to *L. booroolongensis* and *L. wilcoxii* on axis one, which explained 79.5% of the variance (Figure 2). Axis two explained only 1.3% of the variance. Individuals that appeared between the two groups on axis one corresponded to putative hybrids or introgressed individuals (Figure 2).

### 3.3 Accuracy of Morphological Classification

From a total of 126 individuals, 24 (19%) were classified as hybrids according to genotype compared to 17 ± 7 individuals (14% ± 6%; mean ± SD from three validators) based on morphology (Table 1, Figure 2). NewHybrids software classified 72 frogs (57%) as pure *L. booroolongensis* , 30 (24%) as pure *L. wilcoxii* , 2 (2%) as F1 hybrids, 4 (3%) as F2 hybrids, 10 (8%) as backcrosses of F1 hybrids to *L. booroolongensis* and 8 (6%) as backcrosses of F1 hybrids to *L. wilcoxii* (Table 1). Validators classified a comparable proportion of individuals into each class based on morphology (Table 1; χ^2^ = 6.08, df = 5, *p* = 0.299).

**TABLE 1:** Number of frogs (percentage in parentheses) from the New South Wales Central Tablelands (126 frogs in total) assigned to each class (parental *Litoria booroolongensis* ; parental *Litoria wilcoxii* ; F1 hybrid; F2 hybrid; or backcross of F1 hybrid to one of the parentals) based on genetic (NewHybrids classification) and morphological methods.

Class	Genetic classification	Morphological classification
*L. booroolongensis*	72 (57.1)	73 ± 6.2 (57.9 ± 5)
*L. wilcoxii*	30 (23.8)	35.7 ± 3.1 (28.3 ± 2.4)
F1 hybrid	2 (1.6)	3 ± 2.6 (2.4 ± 2.1)
F2 hybrid	4 (3.2)	0.7 ± 1.2 (0.5 ± 0.9)
Backcross of F1 hybrid to *L. booroolongensis*	10 (7.9)	9.3 ± 4.7 (7.4 ± 3.8)
Backcross of F1 hybrid to *L. wilcoxii*	8 (6.3)	4.3 ± 1.2 (3.4 ± 0.9)

*Note:* For morphological classification, mean ± SD is presented based on three independent validators.

**FIGURE 3:**
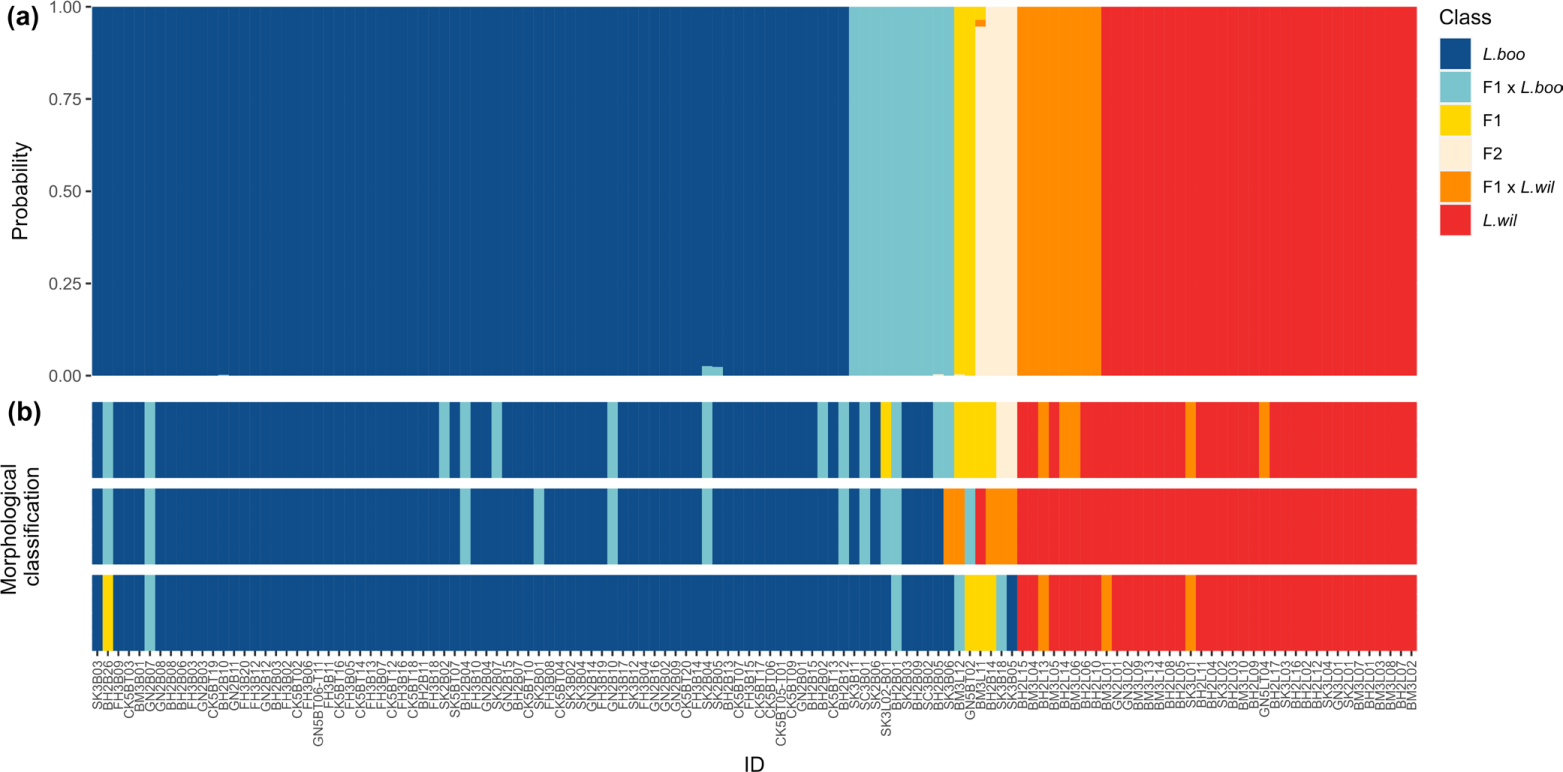
Comparison of species assignments using (a) genetic and (b) morphological methods. Each column represents an individual. (a) NewHybrids classification of each individual with posterior probabilities for given classes (parental *Litoria booroolongensis* (*L. boo*); parental *Litoria wilcoxii* (*L. wil*); F1 hybrids (F1); F2 hybrids (F2); and backcrosses of F1 hybrids to one of the parentals: F1 × *L. boo* and F1 × *L. wil*); (b) species classification based on morphological examination by three independent validators (one row for each validator).

Overall, validators correctly classified 77.8%–81% (mean ± SD: 79.7% ± 1.7%) of frogs based on morphology (Table 2). There were some misidentifications of both pure individuals and hybrids, but morphological classification of pure individuals was more accurate than hybrids (Figure 3, Tables 2 and 3). On average, validators correctly identified 92% (66/72) of the pure *L. booroolongensis* , 96% (29/30) of the pure *L. wilcoxii* and 42% (10/24) of the hybrids (Table 2). Validators varied in their ability to correctly identify hybrids as well as their tendency to suspect hybrids. Of the 24 hybrids that were confirmed by genotype, 42%–71% were misidentified (Table 2). In contrast, validators classified 11, 16 and 25 individuals as putative hybrids (Table 3, Figure 3), thus either underestimating or slightly overestimating the number of hybrids. Of these, 36%–44% were, in fact, genetically pure (i.e., misidentified) (Table 3). Validators were relatively successful at identifying pure species. An average of 91% (range: 88%–93%) and 81% (range: 77%–85%) of individuals classified as putatively pure *L. booroolongensis* and *L. wilcoxii* , respectively, according to morphology were confirmed to be pure by genotype (Table 3).

**TABLE 2:** Number of frogs (percentage in parenthesis) correctly identified by morphology by three validators relative to the number of frogs in each class (based on genotype): Parental *Litoria booroolongensis* (*L. boo*), parental *Litoria wilcoxii* (*L. wil*), F1 hybrid (F1), F2 hybrid (F2) or backcross of F1 hybrid to one of the parentals (F1 × *L. boo* or F1 × *L. wil*).

Validator	All frogs	*L. boo*	*L. wil*	All hybrids	F1	F2	F1 x *L. boo*	F1 x *L. wil*
V1	102/126 (81)	63/72 (87.5)	28/30 (93.3)	14/24 (58.3)	2/2 (100)	2/4 (50)	4/10 (40)	3/8 (37.5)
V2	98/126 (77.8)	65/72 (90.3)	30/30 (100)	9/24 (37.5)	0/2 (0)	0/4 (0)	3/10 (30)	0/8 (0)
V3	101/126 (80.2)	70/72 (97.2)	28/30 (93.3)	7/24 (29.2)	1/2 (50)	0/4 (0)	1/10 (10)	1/8 (12.5)
Mean	100.3/126 (79.7)	66/72 (91.7)	28.7/30 (95.5)	10/24 (41.7)	1/2 (50)	0.7/4 (16.7)	2.7/10 (26.7)	1.3/8 (16.7)

*Note:* When summarizing all hybrids, the F1, F2 and backcross categories were combined.

**TABLE 3:** Accuracy of validators' morphological classifications.

Validator	All frogs	*L. boo*	*L. wil*	All hybrids	F1	F2	F1 x *L. boo*	F1 x *L. wil*
V1	102/126 (81)	63/68 (92.6)	28/33 (84.8)	14/25 (56)	2/5 (40)	2/2 (100)	4/13 (30.8)	3/5 (60)
V2	98/126 (77.8)	65/71 (91.5)	30/39 (76.9)	9/16 (56.2)	—	—	3/11 (27.3)	0/5 (0)
V3	101/126 (80.2)	70/80 (87.5)	28/35 (80)	7/11 (63.6)	1/4 (25)	—	1/4 (25)	1/3 (33.3)
Mean	100.3/126 (79.7)	66/73 (90.5)	28.7/35.7 (80.6)	10/17.3 (58.6)	1.5/4.5 (32.5)	2/2 (100)	2.67/9.3 (27.7)	1.33/4.3 (31.1)

*Note:* Number of frogs (percentage in parenthesis) where validators' morphological species assignments were correct based on genetic data (NewHybrids classification). Possible species assignments were parental *Litoria booroolongensis* (*L. boo*), parental *Litoria wilcoxii* (*L. wil*), F1 hybrid (F1), F2 hybrid (F2) or backcross of F1 hybrid to one of the parentals (F1 × *L. boo* or F1 × *L. wil*). When summarizing all hybrids, F1, F2 and backcrossed individuals were combined.

Hybrids were only detected at sites within Abercrombie National Park and State Conservation Area (GN, SK, BH, SC, BM, Figure 1b), where the species are syntopic. Hybrids comprised 5.3%–100% (median = 31.8%) of the total frogs within these sites (but only two individuals were sampled from site SC, the site with 100% hybrid frequency, due to low frog numbers; Table 4). Genotyping confirmed that only pure *L. booroolongensis* were present at FH and CK (Table 4), consistent with our observations of no *L. wilcoxii* at these sites during our surveys. All validators correctly identified every individual from these sites as pure *L. booroolongensis* (Figure 1b).

**TABLE 4:** Number of frogs (percentage in parentheses) assigned to each class (based on NewHybrids classifications) by survey site.

Site	*L. boo*	*L. wil*	All hybrids	F1	F2	F1 x *L. boo*	F1 x *L. wil*	*N*
BH	12 (36.4)	12 (36.4)	9 (27.3)	—	1 (3)	3 (9.1)	5 (15.2)	33
BM	1 (6.7)	9 (60)	5 (33.4)	1 (6.7)	1 (6.7)	—	3 (20)	15
CK	16 (100)	—	—	—	—	—	—	16
FH	19 (100)	—	—	—	—	—	—	19
GN	14 (73.7)	4 (21.1)	1 (5.3)	1 (5.3)	—	—	—	19
SC	—	—	2 (100)	—	—	2 (100)	—	2
SK	10 (45.5)	5 (22.7)	7 (31.8)	—	2 (9.1)	5 (22.7)	—	22

*Note:* When summarizing all hybrids, the F1, F2 and backcross categories were combined. Abbreviations: F1 × *L. boo*, backcross of F1 hybrid to *L. booroolongensis* ; F1 × *L. wil*, backcross of F1 hybrid to *L. wilcoxii* ; F1, F1 hybrid; F2, F2 hybrid; *L. boo*, *Litoria booroolongensis* ; *L. wil*, *Litoria wilcoxii* ; *N*, sample size.

Success in assigning individuals to specific hybrid classes (F1, F2 or first‐generation backcrosses) based on morphology varied between classes and validators but was low overall (Table 3). Two validators did not classify any individuals as F2 hybrids, and one validator did not classify any individuals as F1 or F2 hybrids (Figure 3). Validators variously classified F1 and F2 hybrid individuals (*n* = 6) as introgressed *L. booroolongensis* , introgressed *L. wilcoxii* , pure *L. wilcoxii* and pure *L. booroolongensis* (Figure 3). Each validator misclassified some introgressed *L. booroolongensis* and *L. wilcoxii* as pure species, but at varying frequencies (Figure 3).

### 3.4 Parental Phenotypes

**FIGURE 4:**
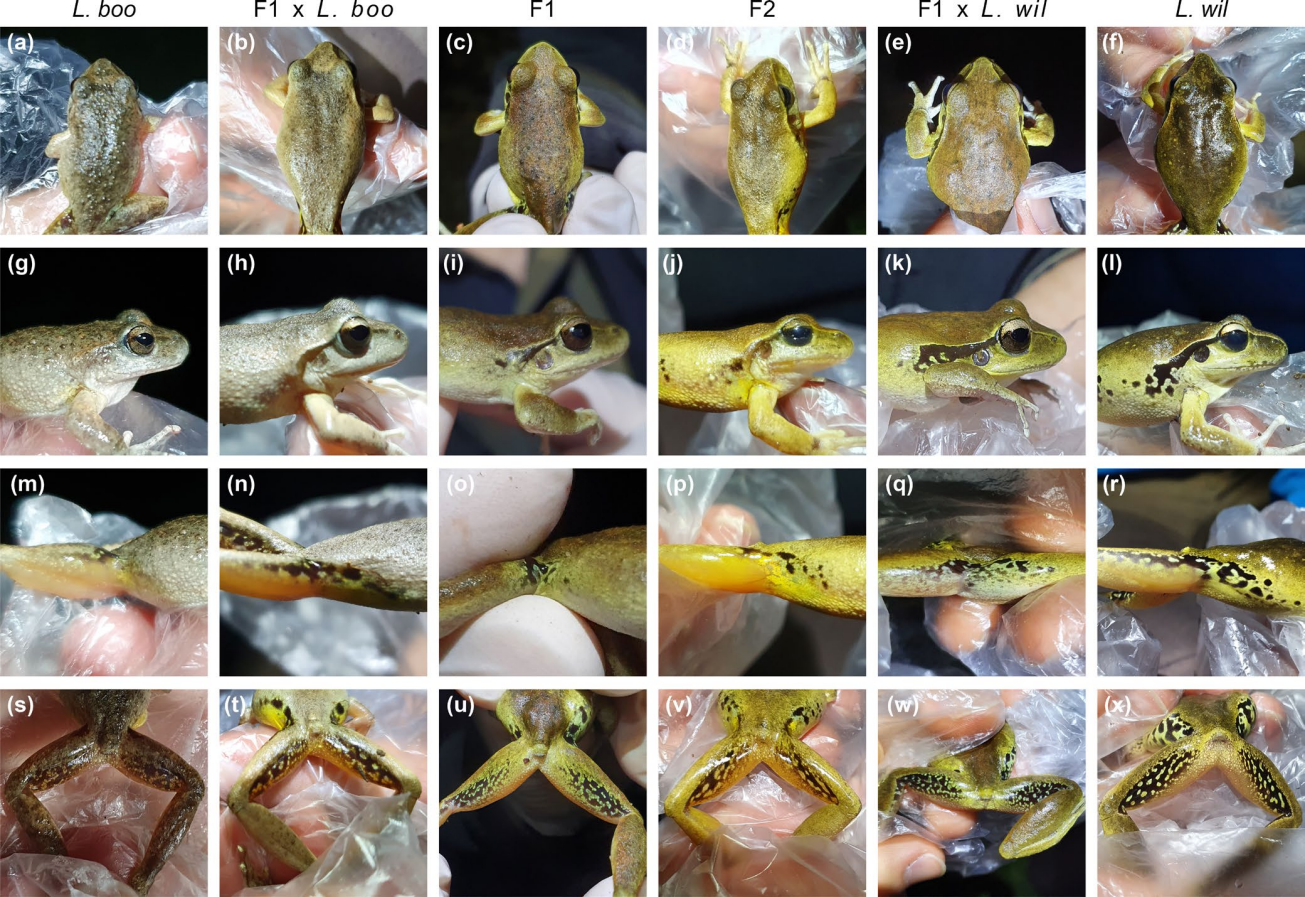
Example morphological characteristics of parental *Litoria booroolongensis* , parental *Litoria wilcoxii* and their hybrids, showing (a–f) dorsum; (g–l) side of face; (m–r) inner thigh; and (s–x) back of thigh. Columns (left to right): Pure *L. booroolongensis* (*L. boo*); backcross of F1 hybrid to *L. booroolongensis* (F1 × *L. boo*); F1 hybrid (F1); F2 hybrid (F2); backcross of F1 hybrid to *L. wilcoxii* (F1 × *L. wil*); pure *L. wilcoxii* (*L. wil*).

While pure *L. booroolongensis* and *L. wilcoxii* were also sometimes misclassified as introgressed individuals based on morphology (Figure 3), the two species generally exhibited diagnosable morphological differences. *Litoria booroolongensis* and *L. wilcoxii* differed most noticeably in size; boldness of pattern and colour on the inner and back of the thigh; facial stripe; and eye colour (Figure 4). *Litoria wilcoxii* were generally larger (longer and heavier) than *L. booroolongensis* (Table S1; Figure S1). The species' traits (Table S2) have been documented previously (Anstis 2017; Clulow and Swan 2018; Cogger 2018), but we found that the most obvious differentiating features of *L. booroolongensis* were a combination of: an indistinct or absent facial stripe (usually continuous, dark and distinct, running from the tip of the snout to above the arm in *L. wilcoxii* ); dark mottling or patches in the inner thigh (usually several distinct black spots or patches in *L. wilcoxii* ); dark brown back of thighs with small lighter coloured spots (usually black back of thighs with more distinct, usually brighter yellow, spots or blotches in *L. wilcoxii* ); gold iris that is brighter in the upper half (gold upper half and dark‐brown lower half in *L. wilcoxii* ); rounded snout (slightly pointed in dorsal view in *L. wilcoxii* ); slight supratympanic ridge (less obvious in *L. wilcoxii* ); different dorsal pattern (dorsum of *L. wilcoxii* typically smoother with fewer or no tubercles); and nearly fully webbed toes (three‐quarters webbed in *L. wilcoxii* ). However, intraspecific morphological variation and overlap in characteristics across species were evident.

### 3.5 Hybrid Phenotypes

Hybrid phenotypes were highly variable, even among individuals belonging to the same hybrid class. Some hybrids closely resembled, or were morphologically indistinguishable from, one of their parental species (e.g., Figure S2) and were misclassified as pure individuals. Others mostly resembled the parental species with the greater genetic contribution but exhibited one or more characteristics typical of the other parental species. For example, some introgressed *L. wilcoxii* had mottled or small patches in the inner thigh (e.g., Figure 4q) but otherwise resembled parental *L. wilcoxii* . Other hybrids exhibited one or more features intermediate between both parental species, such as a visible but indistinct facial stripe (e.g., Figure 4h,j); some dark spots on the inner thigh (e.g., Figure 4o–p); or dark (almost black) backs of thighs with small but distinct lighter coloured spots (e.g., Figure 4u). Mass and SVL of hybrids tended to be intermediate between the parental species (Figure S1).

## 4 Discussion

We have identified a hybrid zone between *Litoria booroolongensis* and *L. wilcoxii* in the NSW Central Tablelands with a relatively high frequency of early generation hybrids (F1, F2 and backcrosses of F1 hybrids to both parental species). We detected hybrids at all five sites where the species co‐occurred in the Central Tablelands, suggesting weak reproductive barriers. As much as 70% of the geographic range of *L. booroolongensis* overlapped with *L. wilcoxii* , indicating potential for considerable interspecific hybridisation and introgression, with uncertain consequences for the persistence of the already threatened *L. booroolongensis* . Complicating matters, hybrids were not reliably identifiable from morphology. These findings highlight the need to use genetic tools to monitor the frequency and extent of hybridisation across species' ranges to appropriately quantify the threats to hybridising species.

Our study adds to the list of Australian frog species known to hybridise in the wild (e.g., Mahony et al. 2021; McDonnell et al. 1978; O'Brien et al. 2018; Parkin et al. 2023; Roberts 2010; Smith et al. 2013) and continues to challenge the notion that hybridisation between species is rare (Mallet 2005). However, the frequency of hybridisation and introgression between *L. booroolongensis* and *L. wilcoxii* in the studied sites in the Central Tablelands (19%; 24/126) appears to be high among frogs (but note the relatively small sample size). The median rate of hybridisation and introgression was 31.8% at sites where both species were detected (5 of 7 sites; range: 5.3–100%). In comparison, across the well‐studied *L. ewingii* – *L. paraewingi* hybrid zone, 10% (49/478) of individuals (4%–16% within sites) were admixed (Smith et al. 2013); *Pseudophryne coriacea* – *P. australis* hybrids comprised 6% (23/371) of the sampled *P. coriacea* population in one breeding site (O'Brien et al. 2018); and *L. myola* – *L. serrata* hybrids comprised only 0%–1.4% and 3.1%–6.8% of 228 and 248 individuals, respectively, in two contact zones (Hoskin 2007; Hoskin et al. 2005).

Disease and the modification and degradation of stream and riparian habitat continue to threaten *L. booroolongensis* (Hunter 2007; Hunter and Smith 2013; TSSC 2021). If their populations were to decline further, or if *L. wilcoxii* abundance increases, the ratio of *L. booroolongensis* to *L. wilcoxii* at sites will decrease, potentially increasing hybridisation rates and the vulnerability of *L. booroolongensis* to demographic or genetic swamping (Field et al. 2008). Habitat modification could also exacerbate hybridisation rates by homogenising habitat, reducing available niches and increasing interspecific interactions (Grabenstein and Taylor 2018).

Demographic swamping may occur if hybridisation rates and outbreeding depression are high (e.g., hybrids are inviable, infertile or have lower mating success than the parental species; Todesco et al. 2016; Wolf et al. 2001). Conversely, genetic swamping may occur if outbreeding depression is low and hybrid population growth exceeds that of one or both parental taxa (Ottenburghs 2021). Based on the presence of F2 hybrids and introgressed individuals in our dataset, it is likely that F1 hybrids are fertile, breeding successfully with each other and with both parental species. This would suggest that hybrids are relatively fit or at least not strongly selected against. Demographic swamping therefore does not appear to threaten either *L. booroolongensis* or *L. wilcoxii* , but it is unclear whether F2 and backcrossed individuals have reduced fitness, and genetic swamping remains a concerning possibility.

The risk of genetic swamping is greatest when there are no reproductive barriers and introgression occurs in the direction of the rare or threatened species (Todesco et al. 2016). Indeed, we detected *L. booroolongensis* – *L. wilcoxii* hybrids in every survey site where the species were syntopic (i.e., GN, SK, BH, SC and BM, Figure 1b), signifying weak pre‐ and/or postzygotic reproductive barriers. The observed patterns of hybridisation might also be related to asymmetry in the reproductive competitive advantages between species (De Sá et al. 2024; Lipshutz et al. 2019). For example, if *L. wilcoxii* males (the larger and more abundant species) have advantages over *L. booroolongensis* males (the smaller, threatened species) during intrasexual disputes (e.g., over territory) or are more attractive to both conspecific and heterospecific females, then hybridisation can continue to occur (De Sá et al. 2024). Whether interspecific mating is intentional (Pfennig 2007) or reflects failed species recognition (e.g., due to similar morphologies, habitat preferences, advertisement calls and overlapping breeding seasons) is unclear, but nonetheless suggests that hybridisation and introgression will likely occur if the parental species have opportunities to interact. There is scope to explore potential barriers to hybridisation such as interspecific differences in behaviour, microhabitat use or advertisement calls. For example, comparative studies of call parameters such as call dominant frequency, number of pulses per note and pulse repetition rate are lacking and opportunities remain to compare the call characteristics of parental individuals and hybrids.

The threat of genetic swamping is significant for *L. booroolongensis* because not only are populations of the species declining, but hybridisation is also potentially occurring across a considerable proportion of its distribution. The distributions of *L. booroolongensis* and *L. wilcoxii* overlap across 70% of the known and predicted geographic range of *L. booroolongensis* (compared to 17% for *L. wilcoxii* ), including much of the Northern Tablelands, raising the possibility of undetected hybridisation in regions not studied here. Future studies could use species distribution models to refine understanding of potential hybrid zones. Regardless, the true overlap (and thus potential for hybridisation) is likely higher as the overlap in the species' ranges includes historical areas of *L. booroolongensis* occupancy (that remain suitable habitat with recolonisation potential; Cutajar et al. 2022b). For example, there are currently less than ten known remaining localities of *L. booroolongensis* in the northern part of its range (NSW OEH 2012; Rowley and Cutajar 2018) and *L. wilcoxii* is present in each, with both species occupying the same habitat during the peak breeding season (P Spark, pers. comm). Radiotracking data has similarly revealed limited niche separation between the two species (Liu 2023). If hybridisation rates in this region are similar to or higher than those observed in the Central Tablelands (i.e., this study), then genetic swamping could be a considerable threat to the already small, vulnerable and genetically divergent northern *L. booroolongensis* population (NSW OEH 2012; Rowley and Cutajar 2018). Furthermore, hybridisation may hinder recolonisation of *L. booroolongensis* as *L. wilcoxii* persists and is common across historic *L. booroolongensis* sites in this region (P Spark, pers. comm).

Advice around hybrid conservation is currently lacking and is discrepant between organisations and countries (Jackiw et al. 2015). Hybrids are ineligible for protection under most conservation policies due to potential negative impacts on species' persistence (Draper et al. 2021). However, introgressive hybridisation can play a key role in evolution (Mallet 2005), which raises important questions about how to best manage hybrid populations between the extremes of neglecting hybrids and affording protection to all. Some guidelines on hybrid management have considered a more balanced approach that centres on the impacts of hybridisation on a case‐by‐case basis, including whether it poses a threat to either parental species and the potential ecosystem‐level outcomes (Allendorf et al. 2001; Jackiw et al. 2015; Ottenburghs 2021). In the case of *L. booroolongensis* – *L. wilcoxii* hybridisation, data on hybrid fitness and hybridisation rates across the range of the species is urgently needed to evaluate the outcomes of hybridisation before making conservation decisions.

Currently, it is unclear how the fitness of hybrids compares to parental species. Further research quantifying and comparing viability, fertility, fecundity, growth, survival, behaviours (e.g., dispersal and habitat use; Coulter et al. 2020; Ellington and Murray 2015; Ficetola et al. 2019) and disease susceptibility of parental species and both early and advanced generation hybrids across various environments (Parris 2001, 2004) will help predict the likely consequences of *L. booroolongensis* – *L. wilcoxii* hybridisation. Conservation policies should not ignore the potential for adaptive hybridisation and introgression. For example, female spadefoot toads facultatively mate with heterospecific males in challenging environmental conditions, improving offspring development rates and survival (Pfennig 2007). However, any positive outcomes of hybridisation should be considered alongside the threat status of the parental species and, in this instance, reflect on whether protecting hybrids will come at the cost of losing pure *L. booroolongensis* .

Monitoring changes to hybrid frequencies over time at different sites across the distribution of *L. booroolongensis* may help determine the risk of genetic swamping. Our results suggest that morphological classification could be useful for this purpose (Table 1), at least as a broad survey tool to identify locations with hybrids, but identifying hybrids at the individual level would require genetic analysis (Tables 2 and 3). Further insights could be gained from assessing historical hybridisation in museum collections and comparing it to current hybridisation dynamics; examining genomic regions exchanged; and correlates of extinction risk (e.g., whether the maternal parent of hybrids is more frequently the rare or more common species (associated with high and low risk, respectively); Todesco et al. 2016; Ottenburghs 2021).

Aside from the broader threat of extinction by hybridisation, the presence of hybrids creates practical challenges for threatened species' management. *Litoria booroolongensis* – *L. wilcoxii* hybrids were often cryptic and undetectable without genotyping, adding uncertainty to morphological classifications that need to be considered when implementing conservation measures. Morphological identification was > 80% accurate for pure *L. booroolongensis* and *L. wilcoxii* (Table 3). While these rates may be suitable for general population monitoring, error rates (i.e., misclassifying hybrids as pure individuals) as high as 20% may be unacceptable for applications such as captive breeding, where uncertain admixture levels could be detrimental to conservation efforts (Costa et al. 2017). The potential for misclassification should be evaluated in line with the need for accuracy and, where high accuracy is important, individuals should be genotyped. For example, genetic screening for hybrids will be important for the recently established captive insurance population of *L. booroolongensis* (DPIE 2021) to ensure that hybrids or introgressed individuals are not unintentionally bred and released back into sites where they may have unintended negative effects.

Morphological identification of *L. booroolongensis* – *L. wilcoxii* hybrids was fraught with errors as phenotype did not reliably reflect hybrid status. Some hybrids were cryptic, whilst others exhibited intermediate morphology or traits of both parental species. As both parental species' phenotypic traits were also highly variable and overlapped, there was no clear delineation between normal intraspecific variation and hybrid phenotypes. Morphological identification of hybrids was therefore inaccurate—up to 44% of suspected hybrids were, in fact, genetically pure (Table 3). For many hybridising species, morphological identification of hybrids can be problematic. For example, many hybrids lack intermediate or transgressive phenotypes and can instead closely resemble one of the parental species (Babik et al. 2003; Majtyka et al. 2022; Neri et al. 2017). However, in some taxa, both cryptic and morphologically intermediate hybrids can exist (Buck et al. 2020; Jasińska et al. 2010). Taken together with these studies, our findings stress that hybrids should not be presumed from morphology. We recommend confirming hybrids with genetic data in any application where hybrid status is important (e.g., for accurate species counts or selecting individuals for breeding programs).

In summary, we detected a relatively high frequency of hybridisation and introgression between the endangered *L. booroolongensis* and its more abundant congener *L. wilcoxii* —two species not examined previously for evidence of hybridisation. Genetic methods will be required to accurately detect hybrids because hybrids were sometimes cryptic. Broadly, our results highlight that hybridisation could be occurring undetected between other related species and could be an unexplored risk to threatened and range‐restricted species. Quantification of the frequency and evaluation of the impacts of hybridisation will be critical for informing appropriate conservation measures for hybridising species.

## Author Contributions

**Gracie Liu:** conceptualization (equal), data curation (lead), formal analysis (lead), funding acquisition (equal), investigation (lead), methodology (equal), visualization (lead), writing – original draft (lead), writing – review and editing (equal). **Jodi J. L. Rowley:** conceptualization (equal), formal analysis (supporting), funding acquisition (equal), investigation (supporting), methodology (equal), supervision (lead), writing – review and editing (equal).

## Conflicts of Interest

The authors declare no conflicts of interest.

## Supporting information


Data S1.

## Data Availability

Data and code to reproduce the analyses are available on GitHub (https://github.com/liugracie/hybridisation_as_a_potential_threat_to_frogs) and are archived in Zenodo (https://doi.org/10.5281/zenodo.14903431).
