# Supplementary material for: Hybridisation as a Potential Extinction Threat to an Endangered Australian Frog
Source: Ecol Evol. 2025 May 29;15(6):e71494. doi: 10.1002/ece3.71494 (PMC12122068; doi:10.1002/ece3.71494)
Supplement: Supplementary file 1 — Data S1. [file ECE3-15-e71494-s001.docx]

**Supplementary Materials**

**Table S1.** Mean and SE snout-vent length (SVL; mm) and mass (g) of adult *L. booroolongensis*, *L. wilcoxii* and their hybrids (F1 hybrids; F2 hybrids; and backcrosses of F1 hybrids to one of the parentals (F1 x *L. booroolongensis* and F1 x *L. wilcoxii*)) by sex (*N* = sample size).

|  |  | SVL (mm) | | Mass (g) | |  |
| --- | --- | --- | --- | --- | --- | --- |
| Class | Sex | Mean | SE | Mean | SE | *N* |
| *L. booroolongensis* | Male | 31.5 | 0.1 | 4.5 | 0.0 | 32 |
|  | Female | 44.2 | 0.2 | 12.2 | 0.2 | 18 |
|  | Unknown | 34.3 | 0.3 | 5.6 | 0.1 | 22 |
| *L. wilcoxii* | Male | 40.3 | 0.1 | 8.0 | 0.1 | 21 |
|  | Female | 45.3 | 1.5 | 11.4 | 1.1 | 3 |
|  | Unknown | 39.3 | 0.5 | 7 | 0.4 | 6 |
| F1 hybrid | Male | 39.0 | 0.7 | 7.0 | 0.7 | 2 |
| F2 hybrid | Male | 37.3 | 0.6 | 5.7 | 0.2 | 4 |
| F1 x *L. booroolongensis* | Male | 33.4 | 0.2 | 4.9 | 0.1 | 9 |
|  | Female | 44 |  | 12.2 |  | 1 |
| F1 x *L. wilcoxii* | Male | 39.3 | 0.8 | 7.5 | 0.3 | 4 |
|  | Female | 41.5 | 0.4 | 8.6 | 0.4 | 2 |
|  | Unknown | 41.5 | 2.5 | 8.3 | 1.8 | 2 |

**Table S2.** Morphological comparison of *Litoria booroolongensis* and *Litoria wilcoxii*. Descriptions were modified from Anstis (2017), Clulow and Swan (2018) and Cogger (2018), based on the morphologies of genotyped individuals*.*

|  | *Litoria booroolongensis* | *Litoria wilcoxii* |
| --- | --- | --- |
| Dorsum | Dull grey to grey-brown or reddish-brown with darker mottling or light brown spots. Skin mostly smooth, sometimes with small scattered low tubercles. | Pale fawn to dark brown, grey-brown, or reddish-brown, sometimes with scattered darker flecks, or irregular dark brown patches and a transverse bar between the eyes. Breeding males can be bright yellow or have yellow limbs and sides of head and body. Skin smooth, occasionally with small, scattered tubercles. |
| Inner thigh | Yellow, sometimes also green or blue-green, with indefinite dark brown or black patches or mottling. Lower half of the front of the thighs pale yellow to orange, almost translucent. | Yellow, usually with several black spots or patches. In some individuals, the groin can be green or blue-green instead of yellow. |
| Back of thighs | Dark brown with small pale spots | Black with scattered yellow, white, blue, or greenish spots and blotches |
| Iris | Gold, with upper half often brighter than lower half | Gold upper half; dark-brown lower half |
| Facial stripe | Usually obscure, but is sometimes visible as a thin, faint stripe from the tip of the snout, through the eye and over the tympanum, to above the arm. | Continuous narrow black stripe from the tip of the snout (usually with a narrow pale border above), through the eye and over the tympanum, to above the arm, where it broadens out and sometimes continues as a series of smaller blotches along the sides of the abdomen. This stripe can be less obvious or absent in yellow breeding males. |
| Tympanum | Distinct with a slight supratympanic ridge | Distinct with pale rim |
| Head shape | Slightly broader than long; snout rounded | Head broad and about as wide as long; snout slightly pointed in dorsal view and rounded in lateral view |
| Ventral surface | Belly white and granular; throat smooth and can be dark in males | Belly white and granular; throat smooth, sometimes with black mottling |
| Webbing | Fingers unwebbed; toes nearly fully webbed | Fingers unwebbed; toes about three-quarters webbed |


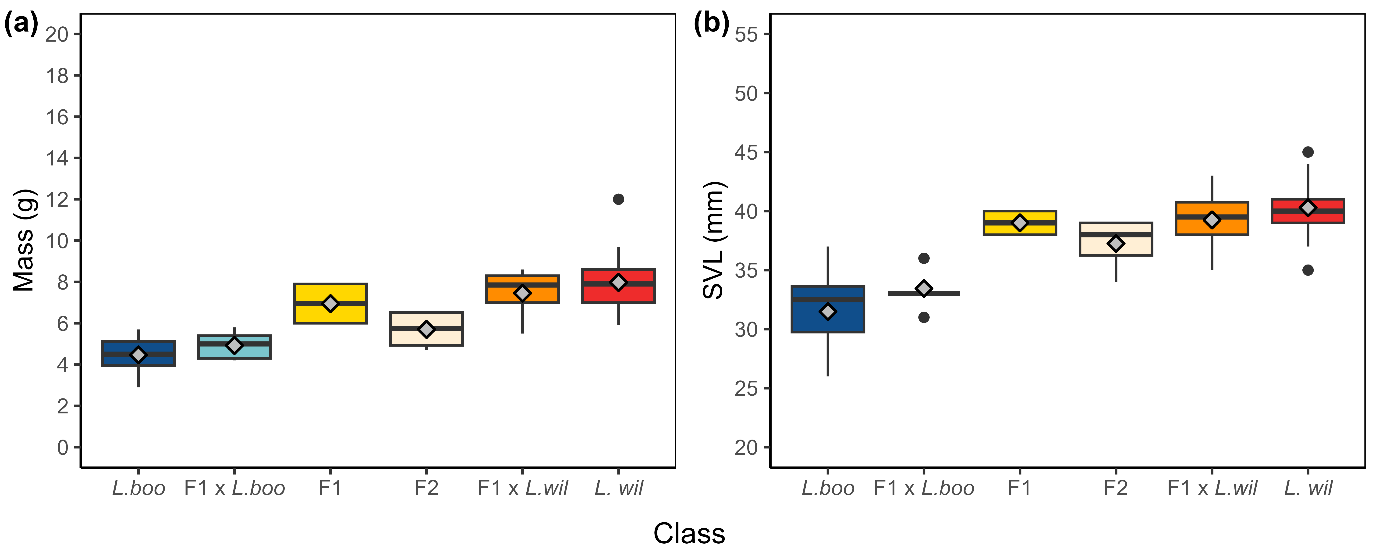


**Figure S1.** Boxplots of (a) mass (g) and (b) snout-vent length (SVL; mm) of adult males in each class: parental *L. booroolongensis* (*L.boo*), parental *L. wilcoxii* (*L.wil*), and their hybrids (F1 = F1 hybrids; F2 = F2 hybrids; F1 x *L.boo* = backcross of F1 hybrid to *L. booroolongensis*; F1 x *L.wil* = backcross of F1 hybrid to *L. wilcoxii*), showing medians, interquartile ranges, and full ranges, overlaid with the mean (grey diamonds).


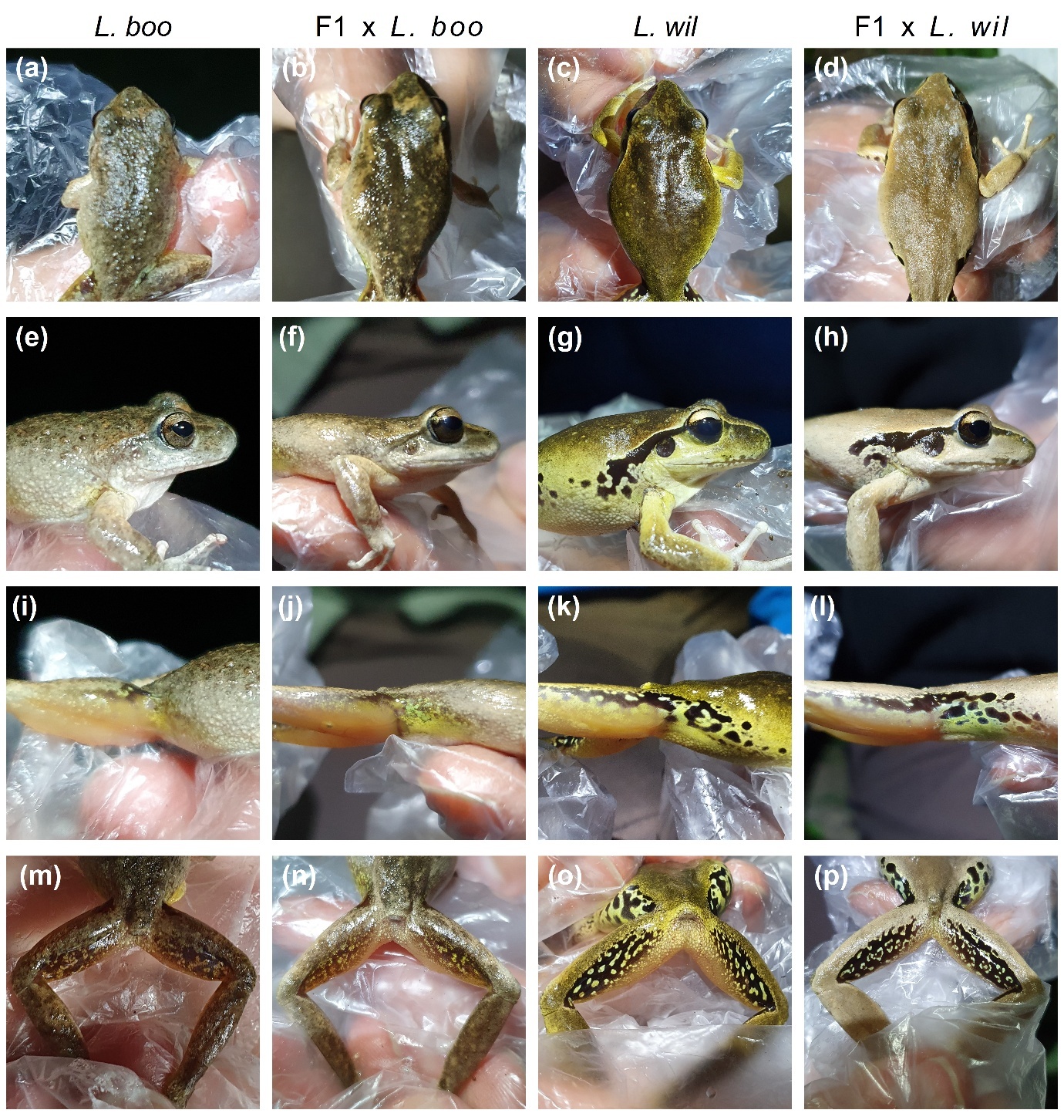


**Figure S2.** Morphological characteristics of parental *Litoria booroolongensis*, parental *Litoria wilcoxii*, and cryptic hybrids, showing (a–d) dorsum; (e–h) side of face; (i–l) inner thigh; and (m–p) back of thigh. Columns (left to right): pure *L. booroolongensis* (*L. boo*); backcross of F1 hybrid to *L. booroolongensis* (F1 x *L. boo*) which was misidentified as a pure *L. booroolongensis* based on morphology; pure *L. wilcoxii* (*L. wil*); backcross of F1 hybrid to *L. wilcoxii* (F1 x *L. wil*) which was misidentified as a pure *L. wilcoxii* based on morphology.
